# Supplementary material for: Thermal control of the topological edge flow in nonlinear photonic lattices
Source: Nat Commun. 2022 Jul 29;13:4393. doi: 10.1038/s41467-022-32069-7 (PMC9338248; doi:10.1038/s41467-022-32069-7)
Supplement: Supplementary file 1 — Supplementary Information [file 41467_2022_32069_MOESM1_ESM.pdf]

Supplementary Information

Thermalized equilibrium states of nonlinear photonic topological insulators

Pawel S. Jung[1,2,†], Georgios G. Pyrialakos[1†], Fan O. Wu[1], Midya Parto[1], Mercedeh Khajavikhan[1,3], Wieslaw Krolikowski[4,5] and Demetrios N. Christodoulides[1]

1. CREOL, College of Optics and Photonics, University of Central Florida, Orlando, Florida 32816, USA
2. Faculty of Physics, Warsaw University of Technology, Koszykowa 75, 00-662 Warsaw, Poland
3. Ming Hsieh Department of Electrical and Computer Engineering,University of Southern California, Los Angeles, California 90089, USA
4. Laser Physics Centre, Research School of Physics and Engineering, Australian National University, Canberra, ACT 0200, Australia
5. Australia5Science Program, Texas A&M University at Qatar, Doha,

Contents

Supplementary Information 1
Supplementary Note I. Weak nonlinear Haldane lattices – internal energy and power 1
Supplementary Note II. The Rayleigh–Jeans distributions and the Equation of State 2
Supplementary Note III. Temperature and chemical potential in the optical systems with and without bandgap 3
Supplementary Note IV. The current in the Haldane lattice in ribbon configuration 5

Supplementary Note I. Weak nonlinear Haldane lattices – internal energy and power

The nonlinear dynamics of a nonlinear Haldane lattice can be described in a more compact form through:

$$i\frac{d}{dt}|\psi\rangle + \left(\hat{H}_L + \hat{H}_{NL}\right)|\psi\rangle = 0 \qquad \text{S1}$$

where, $|\psi\rangle = [a_1, a_2, \dots, a_n, \dots a_M]^T$ is the state vector and $\hat{H}_L$, $\hat{H}_{NL}$ denote the linear and interacting Hamiltonian operator, respectively. In the case of a finite Haldane lattice the linear operator $\hat{H}_L$ is Hermitian and poses a discrete complete set of M orthogonal eigenvectors (modes) $|\Psi_i\rangle$ (i =1.2,…, M) that satisfy the eigenvalue problem $\hat{H}_L|\Psi_\mathrm{i}\rangle = \varepsilon_i|\Psi_\mathrm{i}\rangle$ where ε_i are the corresponding real eigenvalues. Under weak nonlinear conditions, we assume that the evolution is dominated by the linear part of the Hamiltonian and nonlinearity is treated only as the means to allow the redistribution of optical power among all modes in an ergodic fashion. In this respect, the state vector is decomposed in the linear base of $\hat{H}_L$ as a superposition of the eigenvectors

$|\psi(t)\rangle = \sum_i^M c_i(t)|\Psi_i\rangle$, where $c_i(t)$ is the modal occupancies. In this system, the total optical power, given by $= \sum_i^M |c_i|^2$ as well as the expectation value of the linear Hamiltonian $H_L = \langle\psi|\widehat{H}_L|\psi\rangle = \sum_i^M \varepsilon_i |c_i(t)|^2 = U$ are conserved, serving as the first and second invariant of the system. The linear part U can be directly obtained by the initial conditions at $t = 0$ as $U \cong -H_L(t = 0) = -\langle\psi_{(t=0)}|\widehat{H}_L|\psi_{(t=0)}\rangle$.

Supplementary Note II. The Rayleigh–Jeans distributions and the Equation of State

In the microcanonical ensemble (in the context of optics), we consider a conservative system with finite number of modes M having energy levels ε_i, each associated with a degeneracy g_i. Let us assume that the system is composed of $N = \sum_i n_i$ indistinguishable photons n_i at a specific wavelength distributed over g_i states with the same energy ε_i, having total energy ("optical energy") $E = -\sum_i \varepsilon_i n_i$. In such a system, the number of ways (W) in which one can arrange n_i particles within g_i levels is given by

$$W = \prod_i \frac{(n_i + g_i - 1)!}{n_i!\,(g_i - 1)!} \qquad \text{S2}$$

Maximizing the total optical entropy, given by $S_N = \ln W$, under the two individual constrains (N and E), leads to a Bose–Einstein distribution:

$$\frac{n_i}{g_i} = \frac{1}{e^{-\alpha-\beta\varepsilon_i} - 1} \qquad \text{S3}$$

with the help of the Stirling approximation ($\ln n! = n \ln n - n$) and by involving the Lagrange multipliers α, β. Under the condition $-\alpha - \beta\varepsilon_i \ll ,1$ the Bose-Einstein distribution reduces to a Rayleigh–Jeans distribution:

$$\frac{n_i}{g_i} = -\frac{1}{\alpha + \beta\varepsilon_i} \qquad \text{S4}$$

This particular limit can be valid in the context of classical optics, considering that the number of photons (associated with the total power) is much larger the number of available modes ($\frac{n_i}{g_i} \gg 1$ hence $e^{-\alpha-\beta\varepsilon_i} \to 1$). We can then promptly impose the relation $n_i/g_i = n_c|c_i|^2$, where n_c

represents a proportionality factor. To reach the final from of the RJ distribution we adopt the more conventional definitions for optical temperature T and chemical potential μ, as $\alpha = \frac{\mu}{Tn_c}$, $\beta = \frac{1}{Tn_c}$. obtaining:

$$|c_i|^2 = -\frac{T}{\mu + \varepsilon_i} \qquad \text{S5}$$

In a finite optical system that supports M modes we can impose the following definitions, the optical entropy $= \sum_i^M ln|c_i|^2$, the total power $P = \sum_i^M |c_i|^2$ and the optical energy $U = -\sum_i^M \varepsilon_i |c_i|^2$. Taking these expressions into account we may obtain the following expression:

$$\frac{1}{T}U - \frac{\mu}{T}P = \frac{1}{T}\sum_i^M \varepsilon_i |c_i|^2 - \frac{\mu}{T}\sum_i^M |c_i|^2 = \sum_i^M \left(\frac{\varepsilon_i}{\varepsilon_i + \mu} + \frac{\mu}{\varepsilon_i + \mu}\right) = M\,, \qquad \text{S6}$$

which leads to the Equation of state in its final form:

$$U - \mu P = MT. \qquad \text{S7}$$

Supplementary Note III. Temperature and chemical potential in the optical systems with and without bandgap

Any possible optical excitation in a finite system with M number of modes can be associated with a particular power and energy value. The optical temperature T and chemical potential μ can then be uniquely determinate. To do so, we combine the expression of the total power $P = \sum_i^M |c_i|^2$ with eq. (S4) and the equation of state, resulting in

$$P = \sum_i^M -\frac{T}{\varepsilon_i + \mu} = \sum_i^M -\frac{T}{\varepsilon_i + P^{-1}(U - MT)} \qquad \text{S8}$$

which is solved for the temperature T. Subsequently, the chemical potential can be obtained by substituting the value of T into the Equation of state $\mu = \frac{U - MT}{P}$. This process provides a consistent equilibitum solution through eq. (S4) for any excitation in the weak nonlinear regime. Figure S1

demostrates the equilibrium states for single-mode, single-site, non-uniform and quasi-random excitations respectively in a topological Haldane lattice in a finite triangular configuration.

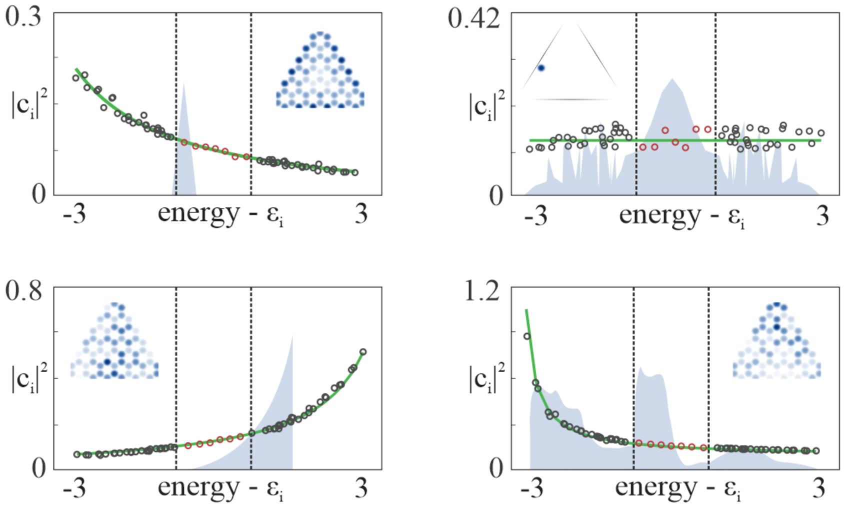

Fig. S1. Equilibrium states for single-mode, single-site, non-uniform and quasi-random excitations respectively in a topological Haldane lattice in a finite triangular configuration. The lattice subfigures depict the initial excitations with a spectrum represented by the shaded regions.

On the other hand, as mentioned in the main manuscript, a bandgap in the optical system can lead to prethermalization. In this case, the temperature and chemical potential can be exclusively predicted by considering each energy group separately. Let's consider a gapped system that supports M eigenmodes with corresponding eigenvalues ε_i, excited with total power $P = \sum_i^M |c_i|^2$ and internal energy $U = -\sum_i^M \varepsilon_i |c_i|^2$. During prethermalization, the two bands exchange optical energy much faster than power and hence the temperature and chemical potential can be deduced by treating separately the lower (l) and upper (u) energy groups. In this respect, let us consider the lower band supporting M_l modes with eigenvalues $\varepsilon_i^{(l)}$ $(i = 1: M_l)$ and the upper band with $M_u = M - M_l$ modes and eigenvalues $\varepsilon_i^{(u)}$ $(i = M_l + 1: M_u)$, respectively. The corresponding optical power and internal energy are $P_l = \sum_i^{M_l} |c_i|^2$, $U_l = -\sum_i^{M_l} \varepsilon_i^{(l)} |c_i|^2$ for the lower band and $P_u = \sum_{M_l+1}^{M_u} |c_i|^2$, $U_u = -\sum_{M_l+1}^{M_u} \varepsilon_i^{(u)} |c_i|^2$ for the upper band. The global and local optical powers and internal energies are related through $P = P_u + P_l$ and $U = U_u + U_l$, respectively. Taking the former expressions into account the optical temperature and chemical potential can be found by solving the following equations:

$$P_l = \sum_{i}^{M_l} -\frac{T_l}{\varepsilon_i + \mu_l} = \sum_{i}^{M_l} -\frac{T_l}{\varepsilon_i + P_l^{-1}(U_l - M_l T_l)};\ \mu_l = \frac{U_l - M_l T_l}{P_l} \qquad \text{S9}$$

$$P_u = \sum_{i=M_l+1}^{M_u} -\frac{T_u}{\varepsilon_i + \mu_u} = \sum_{i=M_l+1}^{M_u} -\frac{T_u}{\varepsilon_i + P_u^{-1}(U_u - M_u T_u)};\ \mu_u = \frac{U_u - M_u T_u}{P_u} \qquad \text{S10}$$

Supplementary Note IV. The current in the Haldane lattice in ribbon configuration

In the ribbon case the currents associated with the fundamental bulk mode (highest-order mode) circulate in small paths, resulting into a 0 net average current (Fig. S2c). At the edges (same figure), this circulation leads to a small unidirectional current which flows in opposite directions between the bottom and top edge. The incoherent summation of all currents from all bulk modes is shown in Fig. S2d. in this case, we see that there are surprisingly zero bulk currents while at the edge channels there is a finite flow with a definite sign and direction. Nonetheless, the net current remains 0 as the top edge channel is balanced by the bottom flow. In Fig. S2d we show that, as expected, the total average current associated with the top edge states flows in the left direction. Similarly, in Fig. S2e the bottom edge states result in currents flowing in the right direction. The summation of the currents from Fig. S2d, Fig. S2e and Fig. S2f gives a net 0 current, both locally and globally. This is why at infinite temperatures where a statistical equipartition of bulk and edge modes occurs, the total edge currents will be zero ($J_B + J_E = 0$). For different temperatures, an imbalance between bulk, top and bottom edge state contributions results in finite net currents.

In the triangular configuration we follow a similar approach in analyzing these currents. In this case there is no bottom and top edge, all edge states are associated with a clockwise circulating net flow. On the other hand, all bulk states are associated with a counterclockwise net flow at the edges (similarly to the comparison between Figs. S2d, S2e and S2f). The average net contribution of either (J_B or J_E will be always net 0 because the structure is now finite, the current cannot escape). Instead, we analyze the net current circulating in the triangular structure by calculating the projection of the currents along the edges of the lattice (in this case in the clockwise direction). These results are shown in Fig.2b (of the main manuscript) for different temperatures.

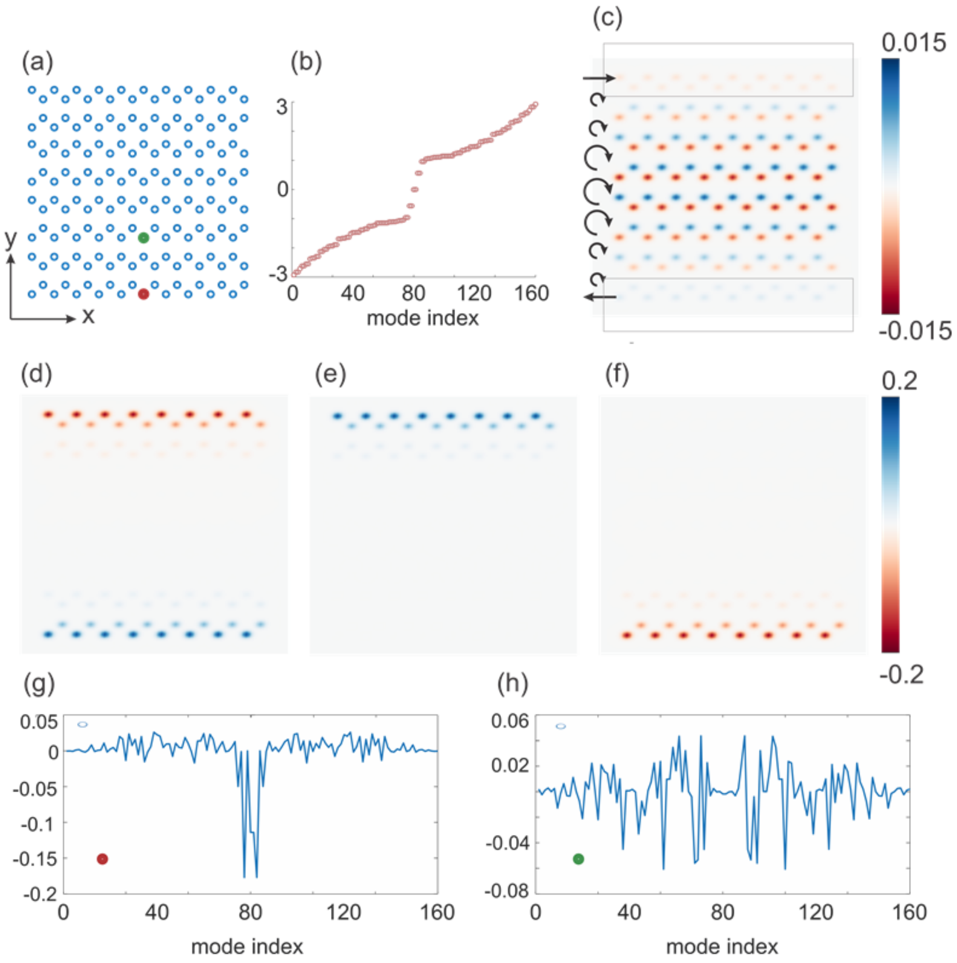

Fig.S2 (a) The Haldane lattice with 160 sites (b) The band structure with parameters (Δ=0,t_1=1,t_2=0.2,φ=π/2) in a ribbon configuration with periodic boundary conditions at the armchair edges, (c) local currents for the fundamental (highest-order) mode. (d-f) Total current contributed by (d) the bulk modes (e) the top edge states (f) the bottom edge states at modal equipartition (g)The local currents at the edge site marked in the red (a) and (h) the local currents at the bulk site marked in green in (a).

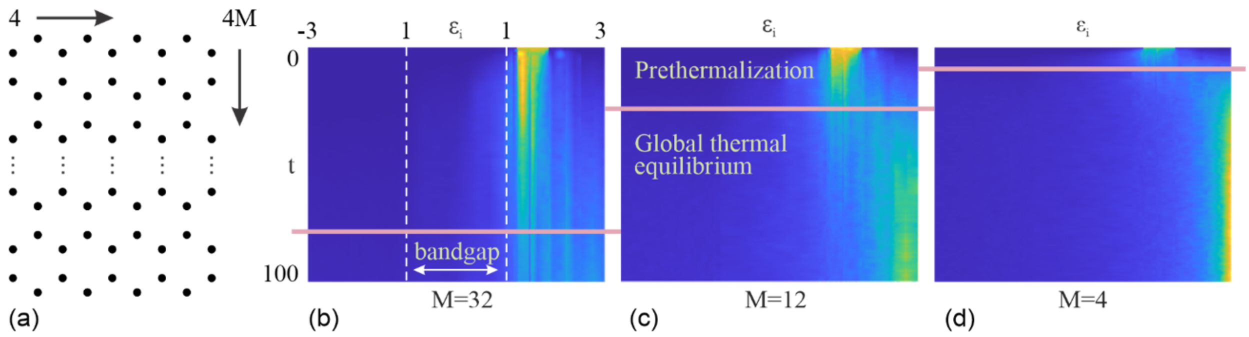

Fig.S3 (a) A Haldane lattice with 4x4M sites and periodic sites in the horizontal direction. Thermal relaxation of light for (b) M=32, (c) M=12 and (d) M=4. As the lattice size increases the edge state's role in suppressing prethermalization is diminished due to their localization effect.
